# Ultra-High-Performance Liquid Chromatography–Tandem Mass Spectrometry and Network Pharmacology Reveal the Mechanisms of *Rhodiola crenulata* in Improving Non-Alcoholic Fatty Liver Disease

**DOI:** 10.3390/cimb47050324

**Published:** 2025-05-01

**Authors:** Xin Zeng, Jianwei Wang, Qinyi Xu, Chengdan Deng, Xi Yi, Shang Wang, Ling Yao, Wei Xiang

**Affiliations:** 1 Chongqing Key Laboratory of Traditional Chinese Medicine for Prevention and Cure of Metabolic Diseases, Chongqing University of Chinese Medicine, Chongqing 402760, China; zeng_xin928@163.com (X.Z.); wjwcq68@163.com (J.W.); dcd0843@163.com (C.D.); wangshang0227@sina.com (S.W.); 2 College of Traditional Chinese Medicine, Chongqing Medical University, Chongqing 400016, China; zsjxqy@126.com (Q.X.); yeexien@163.com (X.Y.)

**Keywords:** *Rhodiola crenulata*, non-alcoholic fatty liver disease, UHPLC-MS, network pharmacology, molecular docking, molecular dynamics simulations

## Abstract

*Rhodiola crenulata* (RC) is a traditional herb and functional food that has demonstrated beneficial effects in improving physical function, enhancing work capacity, alleviating fatigue, and preventing altitude sickness. Additionally, RC has shown promising effects in the treatment of non-alcoholic fatty liver disease (NAFLD), although its specific bioactive components and underlying mechanisms remain unclear. In this study, ultra-high-performance liquid chromatography–mass spectrometry (UHPLC-MS) combined with network pharmacology was employed to identify six potential bioactive compounds from the serum of rats treated with RC—Salidroside, Tyrosol, Crenulatin, Catechin gallate, Eriodictyol, and Rhodiooctanoside—that may contribute to its therapeutic effects on NAFLD. The efficacy of these compounds in improving NAFLD was assessed in vitro using HepG2 cells exposed to Palmitic acid (PA), and it was found that Catechin gallate exhibited a significant effect in reducing lipid accumulation in HepG2 cells. Furthermore, based on network pharmacology predictions, molecular docking studies suggested that the primary targets of Catechin gallate in alleviating fatty liver might include ABCB1, DYRK1A, PGD, and FUT4. Molecular dynamics simulations revealed stable binding interactions between Catechin gallate and these four target proteins. This study clarifies the material basis of RC in the treatment of NAFLD and provides a theoretical foundation for the application of RC and Catechin gallate as functional additives for the management of NAFLD.

## 1. Introduction

Non-alcoholic fatty liver disease (NAFLD), also known as metabolic-associated fatty liver disease (MAFLD), is a condition associated with metabolic syndrome characterized by excessive lipid accumulation in the liver [1,2]. NAFLD begins with hepatic steatosis and can progress to non-alcoholic steatohepatitis (NASH) and hepatic fibrosis (HF) and can even advance to hepatic cirrhosis (HS) and hepatocellular carcinoma (HCC) [3]. NAFLD has become a leading cause of liver disease, with a global prevalence exceeding 25% [4]. This condition poses a significant threat to human health and imposes a substantial economic burden on society. Currently, the only FDA-approved oral medication for the treatment of adult patients with NASH and liver fibrosis is Rezdiffra (active ingredient: Resmetirom), which was approved in March 2024 [5,6]. However, its annual cost may range from $39,600 to $50,100 [7], highlighting the urgent need for more affordable and effective treatments. Plant-derived natural extracts, with their multi-component and multi-target properties, have unique advantages in the management of NAFLD [8,9]. However, the unclear identification of active ingredients and mechanisms has hindered their broader application.

*Rhodiola* species are native to high-altitude regions of Asia, Europe, and the Northern Hemisphere [10]. As a traditional medicinal plant, *Rhodiola* has been used for the treatment of various diseases for centuries [11]. *Rhodiola crenulata* (Hook. f. & Thomson) H. Ohba, a perennial herb of the Crassulaceae family and *Rhodiola* genus, is primarily found in high-altitude regions such as Tibet and Yunnan in China [12]. *Rhodiola crenulata* is a traditional Chinese medicine and food homologous plant with a long history of use [13]. It is also considered a functional food in China and northern Europe [14]. The plant has demonstrated significant effects in enhancing physical function, improving work capacity, alleviating fatigue, and preventing altitude sickness [15,16,17]. Studies have indicated that *Rhodiola* extract significantly increases hepatic glycogen synthesis and inhibits lipogenesis, modulating liver glycogen and lipid metabolism through the AMPK signaling pathway [18,19]. Active components of *Rhodiola* have been shown to alleviate hepatic steatosis in three animal models of non-alcoholic fatty liver disease (NAFLD), including high-fat diet (HFD)-induced obese (DIO) mice, KKAy mice, and HFD plus tetracycline-induced Model-T mice [20]. Previous studies have also suggested that *Rhodiola* extract can improve metabolic disturbances in animal models of metabolic syndrome and type 2 diabetes [21] and may enhance autophagy to ameliorate fructose-induced hepatic steatosis [22]. However, the precise bioactive components and underlying mechanisms of action remain unclear.

Network pharmacology is an interdisciplinary field that integrates systems biology, information networks, computer science, and pharmacology [23]. It offers a systematic and comprehensive approach to understanding the complex network interactions between drugs, targets, and diseases [24], providing a theoretical basis for predicting the target genes and diseases that compound herbal medicines may address [25]. Thus, network pharmacology is often employed to predict the potential mechanisms of action of traditional Chinese medicine (TCM) [11]. Except for those that act directly on the gut or skin surface, most components in TCM are considered to exert their effects after being absorbed into the bloodstream [26]. Therefore, serum pharmacochemistry based on the analysis of blood-absorbed components, combined with network pharmacology, allows for rapid prediction of the potential bioactive substances and their mechanisms of action in TCM [27].

In order to identify the possible active ingredients and mechanisms of action of *Rhodiola crenulata* in improving NAFLD, ultra-high-performance liquid chromatography–mass spectrometry (UHPLC-MS/MS) was used to identify the components of RC that enter the body. This was combined with network pharmacology to predict the potential active components and their targets. Subsequently, a hepatocyte lipid accumulation model was constructed for validation, and computational simulations were performed to predict the possible mechanisms of action of the active components. This research contributes to revealing the material basis and mechanisms of action by which *Rhodiola crenulata* improves NAFLD, providing a theoretical foundation for its effective use in the treatment of NAFLD.

## 2. Materials and Methods

### 2.1. Reagents

Acetonitrile was purchased from Merck (Darmstadt, Germany). Standard compounds, including Crenulatin, Salidroside, Tyrosol, Eriodictyol, and Catechin gallate (>98%), were obtained from TargetMol (Boston, MA, USA). Palmitic acid (PA) was purchased from Kunchuang Biotechnology. All cell culture reagents were obtained from Gibco, and the triglyceride assay kit was purchased from Nanjing Jiancheng Bioengineering Institute. HepG2 cells were purchased from Shanghai Institute of Biochemistry and Cell Biology.

### 2.2. Preparation of Extracts and Medicinal Serum

*Rhodiola crenulata* was sourced from Bozhou Chinese Medicine Exchange (Anhui, China), and the material was from Tibet, China. A total of 100 g of the raw material was weighed and extracted using the method described previously [28]. After the dried material was pulverized, 80% ethanol was used for ultrasonic-assisted extraction (700 W, 30 min). The resulting filtrate was collected and filtered, with the process repeated three times to combine the filtrates. The extract was concentrated under reduced pressure at 50 °C using an R-100 rotary evaporator (Büchi, Flawil, Switzerland), followed by freeze-drying to obtain the extract.

The extract was dissolved in a 0.5% sodium carboxymethyl cellulose solution. After 7 days of acclimatization, rats were orally gavaged with the extract at a dosage of 3 g/kg for 3 consecutive days. On the third day, blood was collected from the retro-orbital sinus at 15, 30, 60, 90, 120, 150, and 180 min after the final gavage to prepare medicinal serum. The control serum was collected from rats gavaged with only 0.5% sodium carboxymethyl cellulose solution and no extract. Serum from 3 rats per group was mixed for pre-treatment and analysis. All animal experiments were conducted in accordance with animal protection, welfare, and ethical guidelines and were approved by the Animal Research Ethics Committee of Chongqing Medical University (IACUC-CQMU), approval number: IACUC-CQMU-2024-0008.

### 2.3. UHPLC-MS/MS Analysis

Equal volumes of medicinal serum from each time point were mixed, and methanol (four times the volume) was added. After thorough mixing, the mixture was centrifuged at 5000× *g* for 10 min. The supernatant was then filtered through a 0.22 μm membrane and subjected to UHPLC-MS/MS analysis. The same method was used for pre-treatment and UHPLC-MS/MS analysis of the RC extract and blank serum.

Ultra-high-performance liquid chromatography was performed using a Dionex UltiMate 3000 system (Thermo, Waltham, MA, USA), coupled with a high-resolution mass spectrometer, TripleTOF 5600 (AB SCIEX). For the chromatographic conditions, the column used was Waters ACQUITY UPLC HSS T3 (1.8 μm, 2.1 × 100 mm), with a column temperature of 40 °C and an injection volume of 2 μL. The mobile phase consisted of 2 mM ammonium acetate in water (A) and acetonitrile (B), with a flow rate of 0.4 mL/min. A gradient elution program was applied as follows: 0–1.5 min, 5% B; 1.5–2.5 min, 5–10% B; 2.5–14 min, 10–40% B; 14–22 min, 40–95% B; 22–25 min, 95% B.

The mass spectrometer operated in negative electrospray ionization (ESI) mode, with data acquisition performed using the IDA function for both precursor and product ion scans. The mass scan range for the first stage was 50–1200 m/z, with a collision energy of 30 eV. The nebulizer gas pressure (GS1) was set to 60 psi, with an auxiliary gas pressure of 60 psi and a curtain gas pressure of 35 psi. The temperature was maintained at 650 °C, and the spray voltage was set to −4000 V.

### 2.4. Network Pharmacology Analysis of Blood-Absorbed Components of Rhodiola crenulata

#### 2.4.1. Identification of Active Components and Target Proteins: Construction of the “Drug-Target” Network

The blood-absorbed components of *Rhodiola crenulata* were considered to be the drug, and non-alcoholic fatty liver disease (NAFLD) was chosen as the disease model for network pharmacology analysis. The chemical structures of the target components were obtained from the PubChem database (https://pubchem.ncbi.nlm.nih.gov/), URL (accessed on 9 April 2024), and the drug targets were predicted using the Swiss Target Prediction database (www.swisstargetprediction.ch). The “Drug–Target” interaction network was constructed using Cytoscape 3.7.1 software.

#### 2.4.2. Intersection of Drug Targets and Disease Targets

Disease targets for NAFLD were collected from the GeneCards database (www.genecards.org/). To minimize interference from targets with low relevance to the disease, only those with a relevance score greater than or equal to the median were selected for further analysis. The drug targets were then intersected with the disease targets to identify potential therapeutic targets for the drug in treating the disease.

#### 2.4.3. Protein–Protein Interaction (PPI) Network

The 98 intersection genes, derived from the potential drug targets for treating NAFLD, were input into the STRING database (Search Tool for the Retrieval of Interacting Genes/Proteins) (https://string-db.org/) for PPI network analysis.

Accessing the STRING database (https://string-db.org/), the “Multiple Proteins” option was selected, and the potential targets for treating NAFLD were input into the “List of names” field. The organism was set to “Homo sapiens” to retrieve the PPI network of protein interactions. After non-interacting proteins were removed, the resulting data were exported. The PPI results were analyzed using Cytoscape 3.7.1, and targets with a degree value greater than or equal to two times the median were defined as core targets, constructing the PPI network diagram.

#### 2.4.4. Construction of the “Drug–Disease–Target” Network

The “Drug–Disease–Target” interaction network was constructed using Cytoscape 3.7.1. The “Network Analyzer” function was applied to obtain the degree values (Degree) of the drug-disease-target interactions, which helped to identify the key components of the drug and the strength of their interactions in the treatment of NAFLD.

#### 2.4.5. GO Biological Enrichment and KEGG Pathway Enrichment Analysis

Gene Ontology (GO) biological enrichment analysis was conducted on the obtained genes using the Metascape database (https://metascape.org/). This included the GO Biological Process, GO Molecular Function, and GO Cellular Component for comprehensive enrichment analysis.

KEGG pathway enrichment analysis was also performed using the Metascape database, where the enriched pathways associated with different targets of the drug in the treatment of disease were visualized in pathway diagrams.

### 2.5. Cell Culture and Cell Viability

HepG2 cells were cultured at 37 °C in a 5% CO_2_ atmosphere with DMEM (Gibco, LOT 8122650) containing 10% fetal bovine serum (HyCyteTM, LOT 230305D1). The cells were passaged when they reached 70–80% confluence.

Cell viability was assessed using the CCK-8 method to determine the effects of the blood-absorbed components on HepG2 cell survival. The cells were seeded at 10,000 cells per well in a 96-well plate and cultured overnight. Different concentrations of drugs (Catechin gallate, Salidroside, Tyrosol, Eriodictyol, Crenulatin) were then added (0, 1, 10, 50, 100 μM), with 5 replicates per group. After 24 h of treatment, the medium was discarded, and 90 μL of DMEM and 10 μL of CCK-8 reagent were added. After incubation for 1 h, the optical density (OD) at 450 nm was measured using a Synergy H1 microplate reader (BioTek, South Burlington, VT, USA), and cell viability was calculated.

### 2.6. TG Content Measurement

HepG2 cells (1 × 10^5^ cells per well) were seeded in 6-well plates, and after the cells reached 70% confluence, 250 μM PA and different concentrations of the drug (0, 5, 10, 20, 50 μmol) were added together for co-treatment for 24 h. After treatment, 100 μL of lysis buffer was added to each well for cell lysis. The cells were scraped off and centrifuged at 3000× *g* for 5 min. The lipid accumulation level in the cells was measured using a Triacylglycerol Assay Kit (Nanjing JianCheng Bio, Nanjing, China). The protein concentration in the lysate was determined using a BCA Protein Assay Kit (Nanjing JianCheng Bio, China). The relative TG content (mg/g protein) was calculated by dividing the amount of TG in the sample (mg) by the total protein amount (g).

### 2.7. Oil Red O Staining

HepG2 cells (5 × 10^4^ cells per well) were seeded in a 12-well plate and treated with 250 μM PA and drugs (20, 50 μmol) for 24 h. After treatment, the cells were fixed with 4% paraformaldehyde for 30 min, followed by pre-differentiation with 60% isopropanol for 5 min. Oil Red O staining was performed for 20 min, followed by hematoxylin staining for the cell nuclei. After washing, we photographed the cellular lipid accumulation under an Olympus BX53F microscope at 100× magnification with bright-field imaging.

### 2.8. Molecular Docking of Potential Targets

The potential targets of the most effective compounds identified from the “Drug–Disease–Target” results were selected, with a probability value > 0.8. The corresponding target protein files were downloaded from the Protein Data Bank (https://www.rcsb.org/RCSB). Molecular docking studies of the target compounds with potential targets were performed using Autodock Vina, following the procedures described in the literature [29]. The target proteins with the highest binding affinities were chosen for further analysis of potential binding sites.

### 2.9. Molecular Dynamics (MD) Simulation

MD simulations were performed using Gromacs 2022 software. The GAFF force field was used for small molecules, and the AMBER14SB force field with the TIP3P water model was applied for proteins. The files of protein and small molecule ligands were merged to build the simulation system for the complex. During the MD simulations, hydrogen bonds were constrained using the LINCS algorithm, and an integration step of 2 fs was used. Electrostatic interactions were calculated using the PME (Particle-Mesh Ewald) method with a cutoff value of 1.2 nm. Non-bonded interactions had a cutoff of 10 Å, and the system was updated every 10 steps. The simulations were conducted at 298 K and 1 bar for 100 ps for NVT and NPT equilibration, followed by a 100 ns MD simulation, with conformational data saved every 10 ps. After the simulations were completed, the trajectories were analyzed using VMD and PyMOL.

### 2.10. Data Analysis

Data processing and analysis were conducted using the previously described methods [30]. In brief, data were analyzed with GraphPad prism (version 9.5.0); one-way analysis of variance (ANOVA) with multiple comparisons (Bonferroni’s correction) was applied for studies with more than two groups to identify differences among groups. All data were expressed as the mean ± standard error. A *p* value less than 0.05 was considered statistically significant.

## 3. Results

### 3.1. Analysis of the Effects of Rhodiola Crenulata Extract on Blood Components (In Vivo)

**Figure 1:**
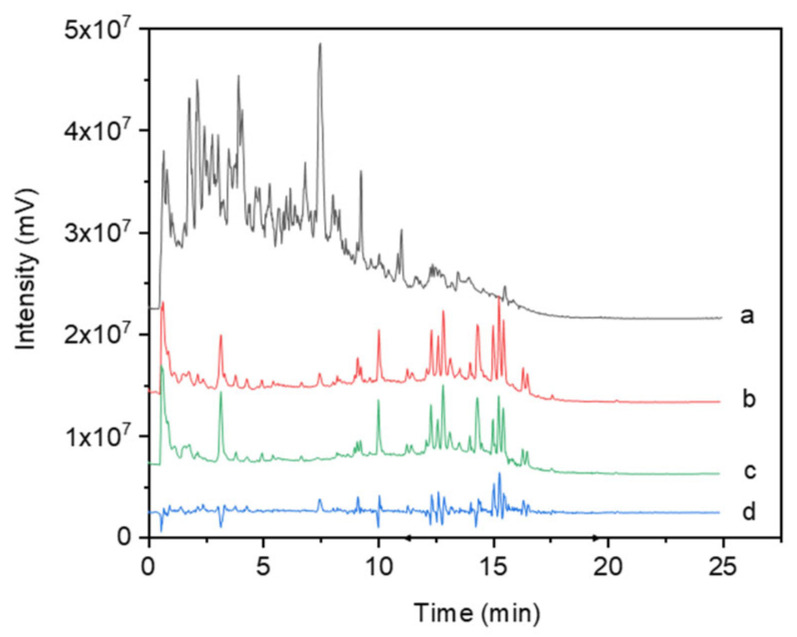
Total ion chromatogram (negative ion mode) of the effect of *Rhodiola crenulata* extract on blood components. a. *Rhodiola* extract; b. Medicinal serum (serum from RC extract-treated rats); c. Blank serum (serum collected from rats gavaged with only 0.5% sodium carboxymethyl cellulose solution and no extract); d. The result obtained after subtracting the control serum from the drug-containing serum.

From the total ion chromatogram (TIC) in negative ion mode of *Rhodiola crenulata* extract and medicinal serum (Figure 1), it can be observed that the extract mainly contains polar components (eluted earlier), with the medicinal serum being similar to the blank serum in terms of major components, though with some minor differences. Through a comparison of the precursor ions and fragment ions in the mass spectrometry data reported in the literature, six components were found to be present in both the extract and medicinal serum but absent in the blank serum: Salidroside, Tyrosol, Crenulatin, Catechin gallate, Eriodictyol, and Rhodiooctanoside. Detailed information on these components is shown in Table 1. These six components were therefore selected for subsequent network pharmacology analysis.

### 3.2. Target Network Analysis

**Figure 2:**
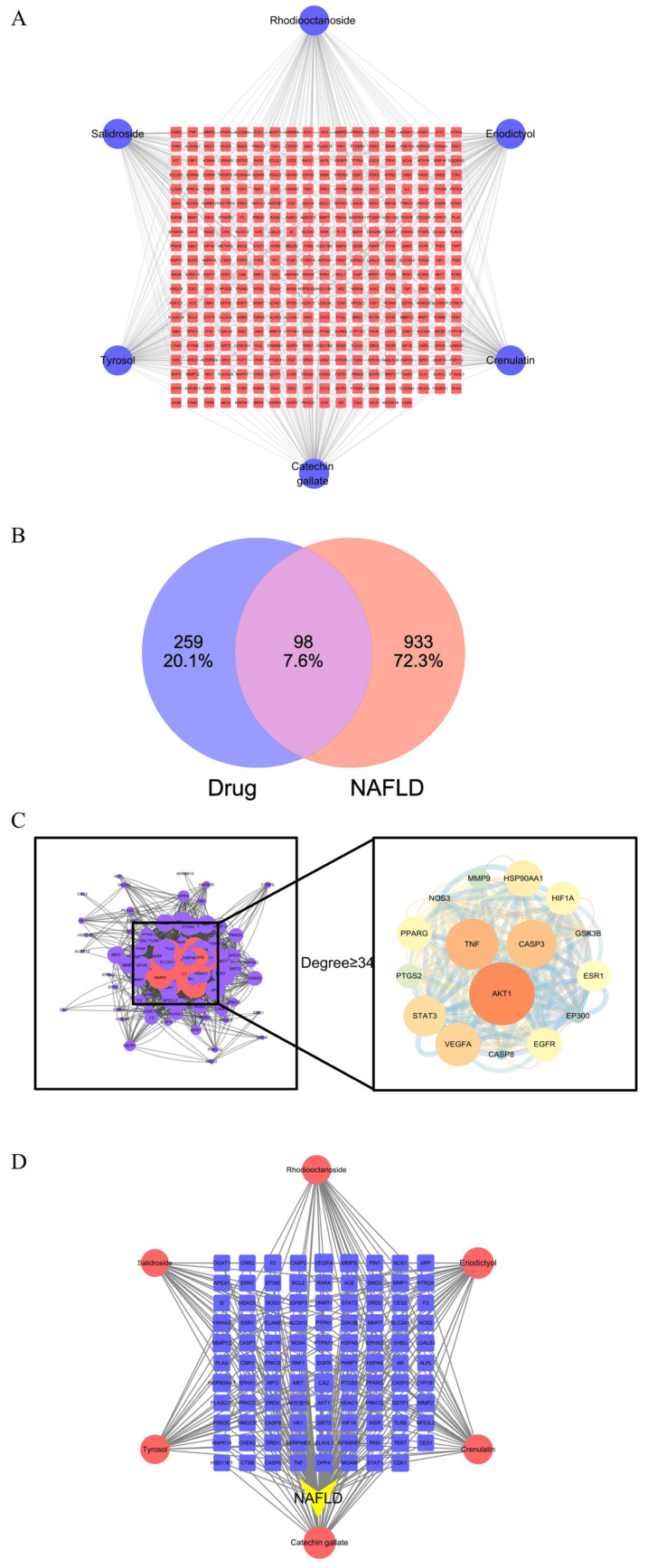
Predicted mechanisms of target components in improving NAFLD. (**A**) “Drug Component–Target” network; (**B**) Venn diagram of drug targets in NAFLD treatment; (**C**) PPI network; (**D**) “Drug Component–Disease–Target” interaction network.

The Swiss Target Prediction database was used to obtain the action targets of the six components, resulting in 357 targets. Based on the active ingredients and their targets, a “Drug Component–Target” network diagram was constructed (Figure 2A), consisting of 363 nodes and 600 edges. In the network, circles represent compounds, and squares represent targets.

NAFLD disease targets were retrieved from the Gene Card database, yielding 2062 targets. After screening, 1031 disease-related targets were selected. The drug targets were intersected with the disease targets, and the resulting 98 potential therapeutic targets were visualized using Cytoscape 3.7.1 (Figure 2B).

The results of the PPI network analysis of the target components in the treatment of non-alcoholic fatty liver disease (NAFLD) are shown in Figure 2C. The node size is determined by the degree value, and the edge thickness corresponds to the combined score. Based on the degree value being greater than or equal to twice the median, 16 core targets were defined: AKT1, TNF, CASP3, VEGFA, STAT3, HSP90AA1, EGFR, PPARG, HIF1A, ESR1, PTGS2, MMP9, EP300, CASP8, NOS3, and GSK3B.

The 98 intersection targets were mapped to the six active compounds, and a “Drug Component–Disease Target” visualization network was constructed (Figure 2D). Among the active compounds, Catechin gallate, Eriodictyol, and Crenulatin ranked highly and are likely to play significant roles in improving NAFLD. The top-ranked targets by degree value are VEGFA, CA2, HSP90AA1, MMP9, MMP2, and MMP13, with degree values of 6, 6, 5, 5, 5, and 5, respectively. This suggests that the drug may exert therapeutic effects on NAFLD by influencing cellular proliferation, angiogenesis, and other processes.

KEGG enrichment analysis of the 98 targets revealed that the treatment of NAFLD is primarily associated with the AGE-RAGE signaling pathway in diabetic complications, HIF-1 signaling pathway, and IL-17 signaling pathway. These findings indicate that multiple components in *Rhodiola* may improve NAFLD through multi-target interactions, and some compounds may have greater activity than Rhodioloside in this regard.

### 3.3. Pathway Enrichment Analysis

#### 3.3.1. GO Biological Enrichment Analysis

**Figure 3:**
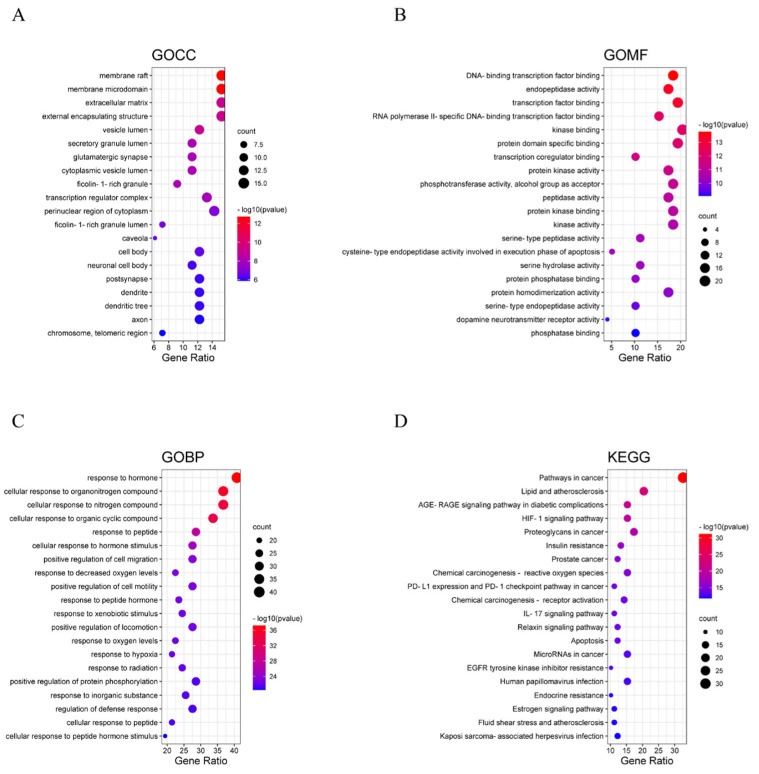
Functional enrichment analysis. (**A**) GO cellular component (GOCC) analysis; (**B**) GO molecular function (GOMF) analysis; (**C**) GO biological process (GOBP) analysis; (**D**) KEGG enrichment analysis.

The 98 genes were imported into the Metascape platform for GO enrichment analysis, which included biological processes (BPs) with 1441 entries, molecular functions (MFs) with 134 entries, and cellular components (CCs) with 87 entries. The top 20 terms for each category were plotted. The biological processes mainly involved responses to hormones (response to hormone), cellular responses to organic nitrogen compounds (cellular response to organonitrogen compound), and cellular responses to nitrogen compounds (cellular response to nitrogen compound) (Figure 3C). The cellular component results included membrane rafts, membrane microdomains, and the extracellular matrix (Figure 3A). Molecular functions mainly encompassed DNA-binding transcription factor binding, endopeptidase activity, and transcription factor binding (Figure 3B).

#### 3.3.2. KEGG Pathway Enrichment Analysis

KEGG pathway enrichment analysis was performed on the 98 targets. Pathways with a *p*-value of less than 0.01 were selected, and the pathways were ordered by ascending *p*-value (*p* < 0.01 is considered significantly enriched, with smaller *p*-values indicating more significant enrichment). The results showed (Figure 3D) that the relevant components in treating NAFLD were mainly associated with the AGE-RAGE signaling pathway in diabetic complications, HIF-1 signaling pathway, and IL-17 signaling pathway. Pathway diagrams were generated. It was found that the drug may act on the AGE-RAGE signaling pathway through 15 targets, on the HIF-1 signaling pathway through 15 targets, and on the IL-17 signaling pathway through 11 targets to exert anti-NAFLD effects.

### 3.4. Effects of Various Components on Hepatic Lipid Accumulation

**Figure 4:**
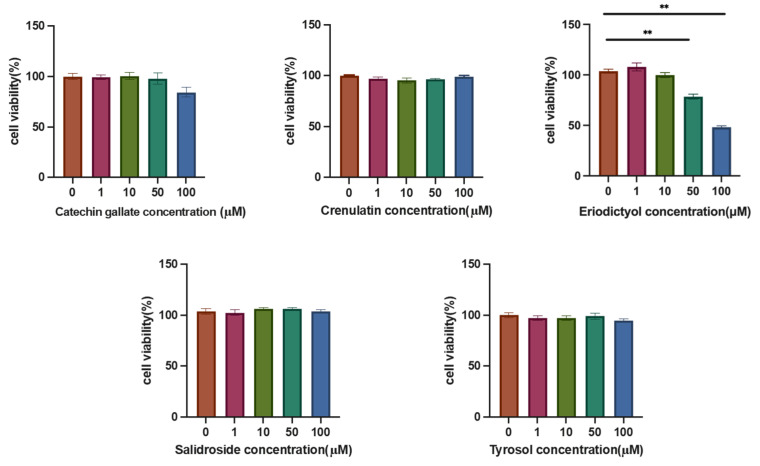
The effect of different components on HepG2 cell viability. ** *p* < 0.01, mean ± SEM, *n* = 3.

To evaluate the effects of the relevant components on lipid accumulation in liver cells, the impact of the compounds on HepG2 cell viability was first assessed. As shown in Figure 4, Crenulatin, Salidroside, and Tyrosol did not affect HepG2 cell viability at concentrations of 100 μM or lower. Catechin gallate had no effect on cell viability at concentrations of 50 μM or lower, but a slight reduction in cell viability was observed at 100 μM. Eriodictyol significantly reduced cell viability at both 50 μM and 100 μM concentrations. Therefore, concentrations of 50 μM or lower were selected for subsequent studies.

**Figure 5:**
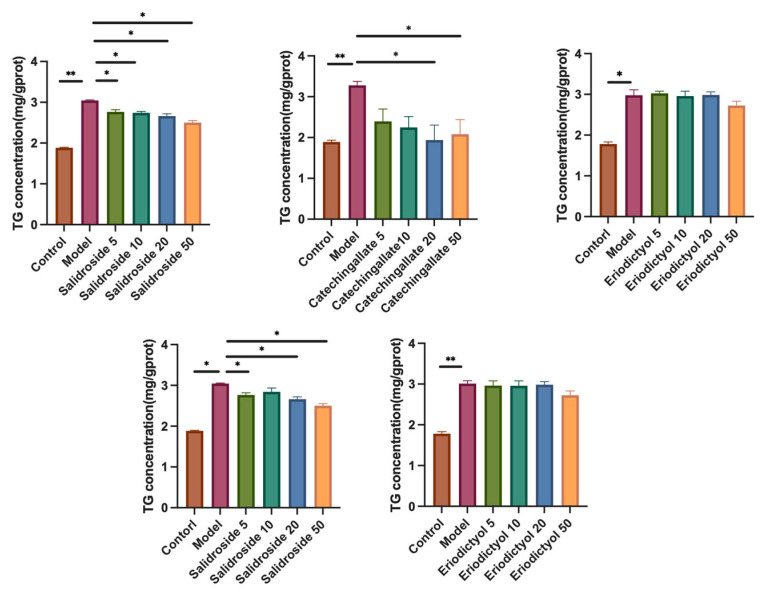
Effects of various components on TG content in HepG2 cells. Cells that were not treated were designated as the Control group, while cells treated with 250 μM PA were designated as the Model group.* *p* < 0.05 and ** *p* < 0.01, mean ± SEM, *n* = 3.

**Figure 6:**
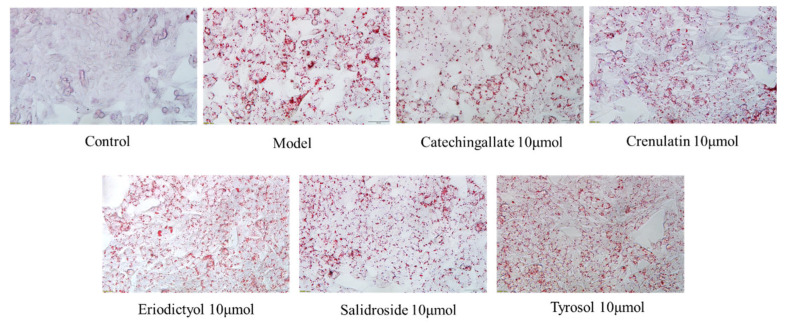
Oil Red O staining of HepG2 cells (100× magnification).

The intracellular TG (triacylglycerol) content reflects lipid accumulation. As shown in Figure 5, in the 250 μM PA-treated model group, the TG content was significantly higher than that in the control group (*p* < 0.01). Catechin gallate significantly reduced the intracellular TG content in HepG2 cells within the concentration range of 5 μM to 50 μM (*p* < 0.05), with the most effective reduction at 20 μM, achieving a decrease of 40.8%. Additionally, Salidroside also significantly reduced the TG content within the concentration range of 5 μM to 50 μM (*p* < 0.01), with the most effective reduction at 50 μM, achieving a decrease of 17.8%. At the same concentration, Catechin gallate exhibited a stronger effect in improving hepatic lipid accumulation compared to Salidroside, while Crenulatin, Eriodictyol, and Tyrosol showed no significant effects on reducing hepatic lipid accumulation. The Oil Red O staining results (Figure 6) corroborated these findings.

### 3.5. Molecular Docking of Catechin Gallate with Core Targets

**Figure 7:**
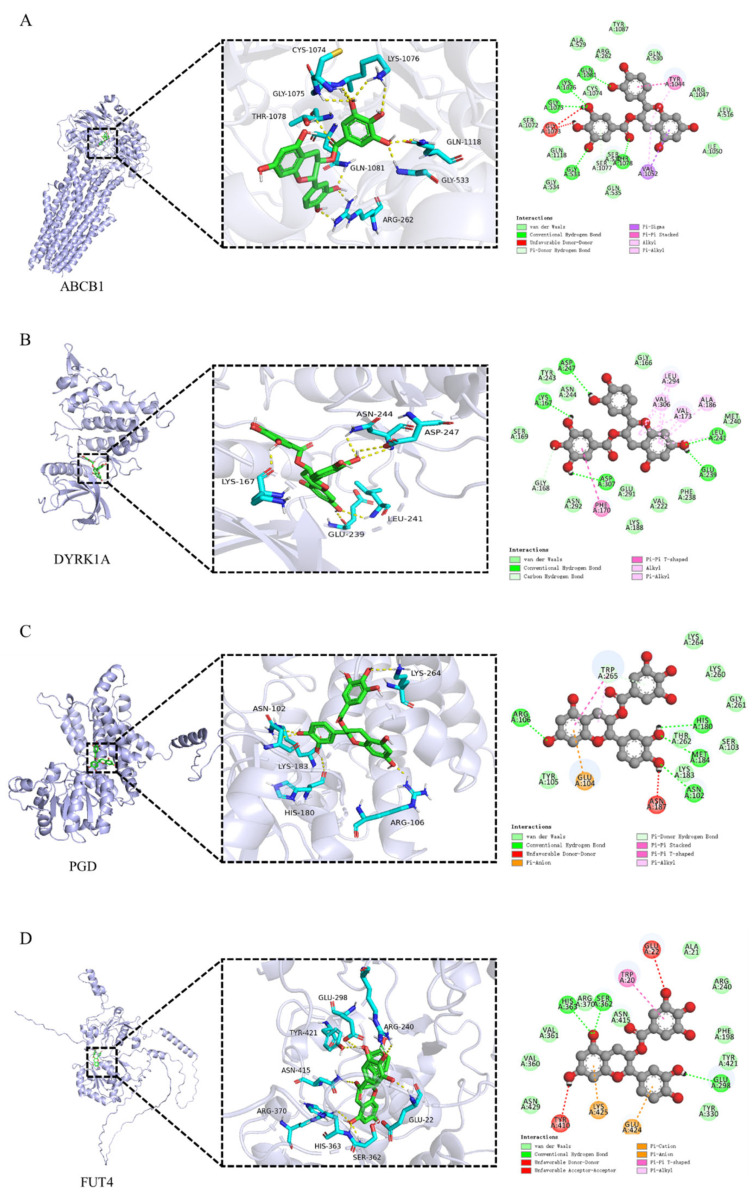
Visualization of molecular docking. (**A**), Catechin gallate-ABCB1; (**B**), Catechin gallate-DYRK1A; (**C**), Catechin gallate-PGD; (**D**), Catechin gallate-FUT4.

To explore the potential mechanism by which Catechin gallate improves lipid accumulation in liver cells, molecular docking studies were performed based on the potential targets of Catechin gallate in the treatment of NAFLD identified through network pharmacology (Supplementary Table S1). Targets with a probability value greater than 0.8 were selected for molecular docking analysis. Since no crystal structures are available for ST3GAL3, FUT7, and FUT4, protein structures predicted by α-fold were used for docking simulations. The results (Table 2) indicate that Catechin gallate exhibits strong binding affinity with ABCB1, DYRK1A, PGD, and FUT4 proteins, with binding energies of −10.5, −9.6, −9.2, and −9 kJ/mol, respectively. Figure 7 illustrates the binding modes of Catechin gallate with these four targets. As shown in Figure 7A, Catechin gallate forms conventional hydrogen bond interactions with the amino acid residues GLY-533, GLY-1075, LYS-1076, THR-1078, and GLN-1081 in the ABCB1 protein and a Pi-donor hydrogen bond interaction with SER-1077. The phenolic hydroxyl group of Catechin gallate interacts with LYS-167, GLU-239, LEU-241, ASP-247, and ASP-307 in DYRK1A through conventional hydrogen bonds, while also forming a carbon–hydrogen bond with GLY-168. Furthermore, the benzene ring of the gallic acid part forms a Pi–Pi T-shaped interaction with PHE-170 (Figure 7B). In Figure 7C, Catechin gallate’s phenolic hydroxyl group forms conventional hydrogen bond interactions with ASN-102, ARG-106, HIS-180, and MET-184 residues in PGD, while also forming multiple interactions with TRP-265, including Pi-donor hydrogen bonds, Pi–Pi T-shaped, and Pi–alkyl interactions. Lastly, Catechin gallate forms conventional hydrogen bond interactions with GLU-298, SER-362, and HIS-363 in FUT4 and also engages in Pi–cation interactions with LYS-425 and GLU-424, as well as a Pi–Pi T-shaped interaction with TRP-20.

### 3.6. Molecular Dynamics Simulation Results

**Figure 8:**
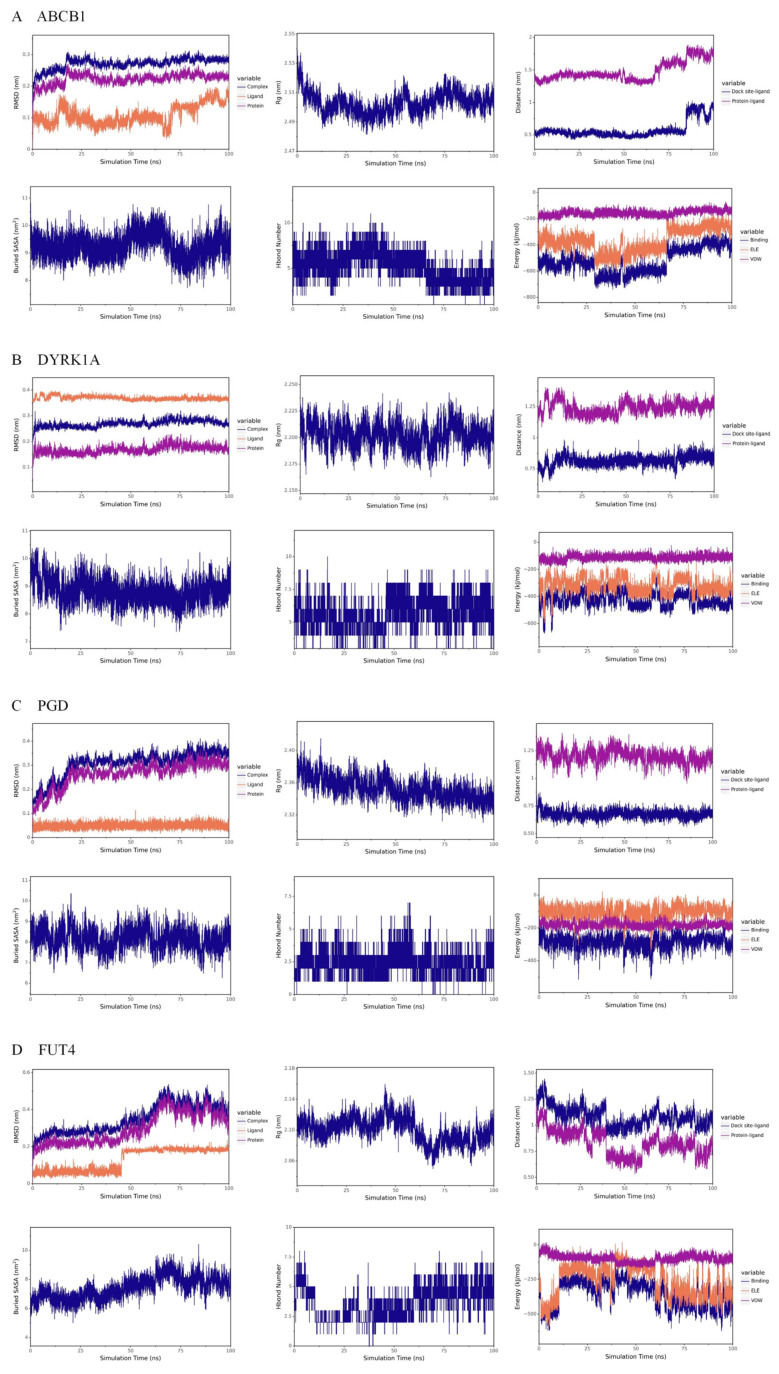
Molecular dynamics simulation results. (**A**), Catechin gallate-ABCB1; (**B**), Catechin gallate-DYRK1A; (**C**), Catechin gallate-PGD; (**D**), Catechin gallate-FUT4.

Root Mean Square Deviation (RMSD) is a commonly used metric to assess the structural differences between two molecules. A lower RMSD indicates that the two structures are more similar. As shown in Figure 8, the RMSD of the Catechin gallate–protein complex and the related protein gradually stabilize during the simulation, indicating that the complex structure becomes more stable over time. The Radius of Gyration (Rg) is an important parameter for assessing the overall compactness of a protein–small molecule complex. A smaller Rg indicates a more compact structure, while a larger Rg suggests a more loosely packed structure. From the figure, it can be observed that the Rg of the Catechin gallate-protein complex gradually decreases and reaches a more stable trend, suggesting that the complex structure becomes more compact and stable as the simulation progresses. The Buried Solvent Accessible Surface Area (Buried SASA) is a parameter that reflects the strength of interactions between molecules; the larger the Buried SASA, the stronger the interaction and the greater the contact area. In Figure 8, the Buried SASA remains relatively stable, indicating that the contact area between the small molecule and the protein is stable, and their binding remains consistent. Hydrogen bonds are key interactions involved in the binding of proteins and ligands. These bonds are related to electrostatic interactions and can reflect the strength of electrostatic forces between the molecules. As shown in Figure 8, the number of hydrogen bonds between Catechin gallate and ABCB1, DYRK1A, PGD, and FUT4 fluctuates between 4–7, 5–7, 1–3, and 2–5, respectively. In the results of van der Waals (VDW) and electrostatic (ELE) interactions between Catechin gallate and the four proteins, VDW represents van der Waals forces and hydrophobic interactions, while ELE refers to electrostatic interactions. “Binding” refers to the sum of the VDW and ELE interactions, representing the binding energy of the small molecule with the protein without considering the solvation effect. In the Catechin gallate–ABCB1 complex, the VDW interaction is relatively more stable than the ELE interaction, and ELE shows periodic fluctuations, which are due to conformational adjustments of the small molecule. The binding energy gradually stabilizes as the simulation progresses, indicating that the interaction between the small molecule and protein becomes more stable over time. In the Catechin gallate–DYRK1A complex, the VDW interaction is also more stable than the ELE interaction, with the ELE interaction remaining relatively stable, indicating that the binding between the small molecule and protein remains steady. For the Catechin gallate–PGD complex, both the VDW and ELE interactions gradually stabilize, suggesting that the binding between the small molecule and protein remains stable. In the Catechin gallate–FUT4 complex, VDW interactions are more stable than ELE interactions, and the ELE interaction gradually stabilizes, indicating that the binding between the small molecule and protein becomes more stable over time. Overall, these results suggest that Catechin gallate forms stable complex structures with ABCB1, DYRK1A, PGD, and FUT4 proteins.

## 4. Discussion

Various compounds have been isolated from *Rhodiola crenulata* extract, with salidroside, tyrosol, and rosavin being its major pharmacologically active components. Other compounds, such as quercetin, rhodiolin, and kaempferol glycosides, have also been identified. Salidroside, one of the primary active ingredients in *Rhodiola crenulata*, has been extensively studied for its role in improving non-alcoholic fatty liver disease (NAFLD). Research indicates that it improves NAFLD by inhibiting hepatic fat synthesis and promoting fatty acid oxidation [34], as well as by regulating gut microbiota to alleviate hepatic steatosis [35]. However, the effects of other components in *Rhodiola crenulata* on NAFLD are still not well understood. In this study, we found that Catechin gallate, under the same concentration conditions, had a stronger effect in improving hepatic lipid accumulation compared to Salidroside. Catechin gallate shows promising potential for the treatment of non-alcoholic fatty liver disease, though its efficacy still needs to be further validated at the animal level.

In this study, through network pharmacology and molecular docking, we identified potential targets for Catechin gallate in the treatment of fatty liver disease, which include ABCB1, DYRK1A, PGD, and FUT4. Among these, PGD exhibited the highest probability value, and molecular docking revealed that the binding affinity between Catechin gallate and ABCB1 was the strongest. 6-Phosphogluconate dehydrogenase (PGD) is a key enzyme in the pentose phosphate pathway (PPP), primarily catalyzing the conversion of 6-phosphogluconate to ribulose 5-phosphate. PGD plays a crucial role in the generation of NADPH [36], which has various cellular functions, including involvement in fatty acid synthesis. Therefore, PGD is an enzyme related to lipid biosynthesis [37,38]. Catechin gallate may improve hepatic lipid production by inhibiting ABCB1. ATP-binding cassette subfamily B member 1 (ABCB1) is closely related to drug absorption [39], and the strong binding affinity between Catechin gallate and ABCB1 (−10.5 kJ/mol) suggests that it can be efficiently absorbed into the body to exert its pharmacological effects. This could explain why Catechin gallate can be readily detected in vivo.

DYRK1A is an evolutionarily conserved protein kinase that plays a significant role in various biological processes [40]. DYRK1A is involved in the progression of several diseases, such as diabetes, Down syndrome, Alzheimer’s disease, and cancer [41,42]. Additionally, Fucosyltransferase 4 (FUT4) promotes cancer progression by enhancing tumor proliferation and migration [43,44]. Inhibition of FUT4 can reduce the proliferation, invasion, and migration abilities of tumors [45,46]. This study suggests that Catechin gallate may have a strong binding affinity for DYRK1A and FUT4, indicating its potential for treating other diseases, such as cancer and neurodegenerative disorders.

Catechin gallate is a compound belonging to the catechin family, which is widely found in various plants, including green tea and cocoa [47]. Among these compounds, Epigallocatechin-3-gallate (EGCG) has been the most extensively studied. EGCG has been shown to improve fatty liver disease through various mechanisms, including regulating liver macrophage polarization [48], altering bile acid metabolism [49], modulating the ROS/MAPK signaling pathway to inhibit apoptosis and promote autophagy [50], and more [51,52]. The structure of Catechin gallate is very similar to that of EGCG, differing by only one phenolic hydroxyl group. It also demonstrates significant potential for improving fatty liver. Future research could focus on the structure–activity relationship of similar compounds, which may help identify even more effective components for improving NAFLD.

## 5. Conclusions

In this study, ultra-high-performance liquid chromatography coupled with mass spectrometry, along with network pharmacology, was used to identify six active components in *Rhodiola crenulata* that may contribute to the improvement of non-alcoholic fatty liver disease (NAFLD): Salidroside, Tyrosol, Crenulatin, Catechin gallate, Eriodictyol, and Rhodiooctanoside. Further investigation using in vitro cell models revealed that Catechin gallate significantly improves lipid accumulation in liver cells. Additionally, molecular docking, guided by network pharmacology results, suggests that Catechin gallate may improve fatty liver through its interactions with ABCB1, DYRK1A, PGD, and FUT4. Molecular dynamics simulations confirmed that Catechin gallate forms stable complexes with these four proteins. These findings suggest that Catechin gallate is a key active component in *Rhodiola crenulata* for improving NAFLD, potentially acting through multiple mechanisms involving ABCB1, DYRK1A, PGD, and FUT4. However, its effects at the animal level and its exact mechanisms still require further investigation.

## Figures and Tables

**Table 1:** Data on the blood components constituting the *Rhodiola crenulata* extract.

Time	Name	Molecular Formula	[M−H]^−^	Ms/Ms	Ref.
1.78	Salidroside	C_14_H_20_O_7_	299.1130	203.0832, 119.0515, 89.0247, 71.0148	[12,31]
1.99	Tyrosol	C_8_H_10_O_2_	137.0260	93.0350	[12]
2.15	Crenulatin	C_11_H_20_O_6_	247.1196	167.0762, 101.0255, 85.0293	[12]
3.90	Catechin gallate	C_22_H_18_O_10_	441.1007	289.0736, 245.0763, 169.0125, 125.0227	[31]
6.43	Eriodictyol	C_15_H_12_O_6_	287.0537	151.0043, 96.9618	[32]
7.46	Rhodiooctanoside	C_19_H_36_O_10_	423.2205	291.1818, 161.0476, 101.0245, 89.0239, 59.0135	[33]

**Table 2:** Molecular docking results of *Catechin gallate* with various targets.

Target	Common Name	Target Class	Probability	PDB ID	Binding Energy
6-phosphogluconate dehydrogenase	PGD	Enzyme	0.999640903	4GWG	−9.2
Beta-secretase 1	BACE1	Protease	0.999640903	1TQF	−7.5
Apoptosis regulator Bcl-2	BCL2	Other ion channel	0.928564807	1G5M	−8
MAP kinase p38 alpha	MAPK14	Kinase	0.895379597	1A9U	−8.6
Microtubule-associated protein tau	MAPT	Unclassified protein	0.862200422	2MZ7	−5.9
DNA (cytosine-5)-methyltransferase 1	DNMT1	Writer	0.862200422	3PTA	−8.6
Dual-specificity tyrosine-phosphorylation regulated kinase 1A	DYRK1A	Kinase	0.862200422	4YLK	−9.6
Beta amyloid A4 protein	APP	Membrane receptor	0.862200422	1AAP	−7.7
Telomerase reverse transcriptase	TERT	Enzyme	0.862200422	5UGW	−7.4
Matrix metalloproteinase 2	MMP2	Protease	0.862200422	1CK7	−8.4
Hepatocyte growth factor receptor	MET	Kinase	0.862200422	1R0P	−8.6
Matrix metalloproteinase 14	MMP14	Protease	0.862200422	3MA2	−8.1
P-glycoprotein 1	ABCB1	Primary active transporter	0.862200422	6C0V	−10.5
Signal transducer and activator of transcription 1-alpha/beta	STAT1	Transcription factor	0.862200422	7NUF	−8.7
Squalene monooxygenase (by homology)	SQLE	Enzyme	0.862200422	6C6N	−8
CMP-N-acetylneuraminate-beta-1,4-galactoside alpha-2,3-sialyltransferase	ST3GAL3	Transferase	0.812371847	α-Fold	−8.5
Alpha-(1,3)-fucosyltransferase 7	FUT7	Transferase	0.812371847	α-Fold	−8.1
Fucosyltransferase 4	FUT4	Enzyme	0.812371847	α-Fold	−9

## Data Availability

The original contributions presented in the study are included in the article/Supplementary Materials, further inquiries can be directed to the corresponding authors.

## Supplementary Materials

The following supporting information can be downloaded at: https://www.mdpi.com/article/10.3390/cimb47050324/s1.
