# Supplementary figures and images for: Ultra-High-Performance Liquid Chromatography–Tandem Mass Spectrometry and Network Pharmacology Reveal the Mechanisms of Rhodiola crenulata in Improving Non-Alcoholic Fatty Liver Disease
Source: Curr Issues Mol Biol. 2025 May 1;47(5):324. doi: 10.3390/cimb47050324 (PMC12110739; doi:10.3390/cimb47050324)

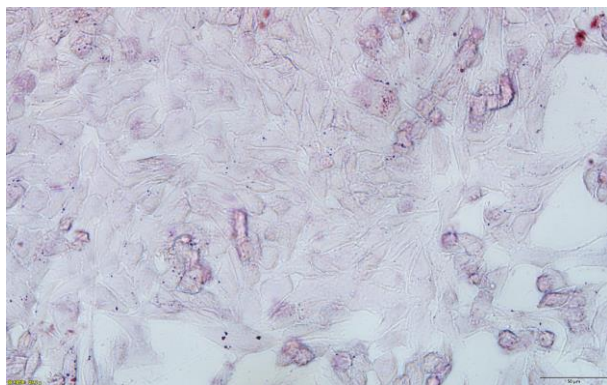

Control

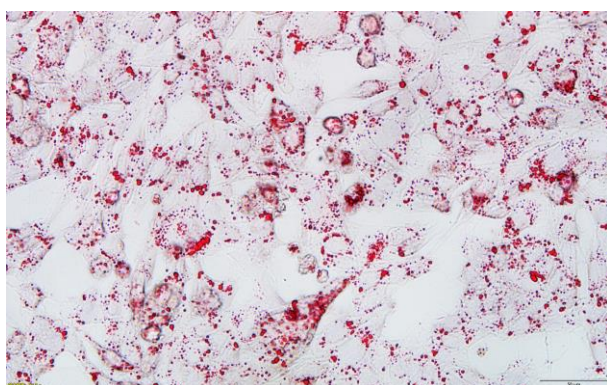

Model

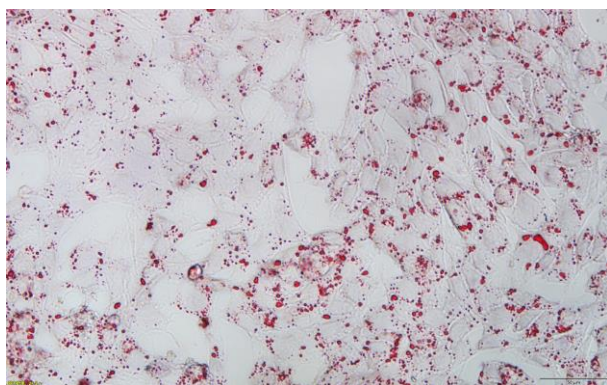

Catechingallate

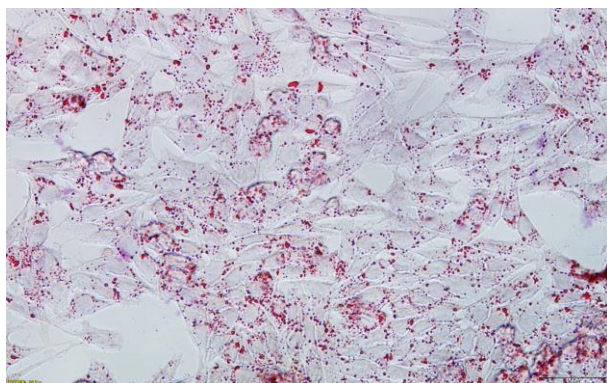

Crenulatin

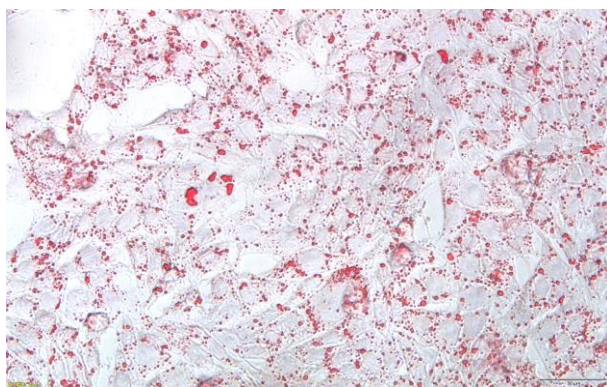

Eriodictyol

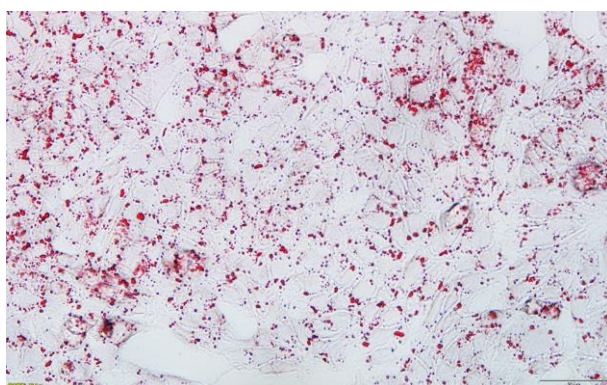

Salidroside

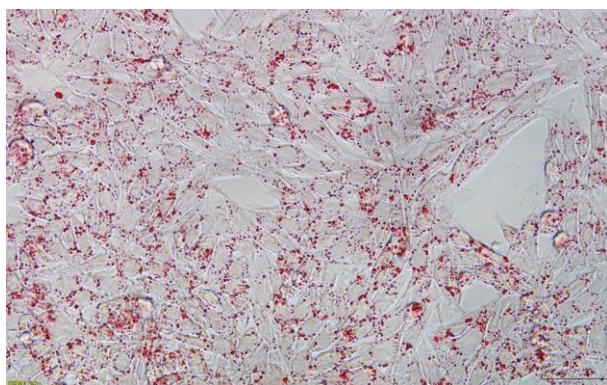

Tyrosol

Supplement: Supplementary file 1 [file cimb-47-00324-s001.zip › Figure 6.pdf]
